# Supplementary material for: Insights into the Possible Molecular Mechanisms of Resistance to PARP Inhibitors
Source: Cancers (Basel). 2022 Jun 5;14(11):2804. doi: 10.3390/cancers14112804 (PMC9179506; doi:10.3390/cancers14112804)
Supplement: Supplementary file 1 [file cancers-14-02804-s001.zip › cancers-1751125-supplementary.pdf]

Table S1. Ongoing (Recruiting and Active not recruiting) clinical trials investigating the combina-tion of PARPi with immunotherapies and other agents (all accessed on 2 June 2022).

	Title	Status	Study Results	Conditions	Interventions
1	Study of CYH33 in Combination With Olaparib an Oral PARP Inhibitor in Patients With Advanced Solid Tumors.	Recruiting	No Results Available	•Ovarian Cancer<br>•Breast Cancer<br>•Solid Tumor<br>•Prostate Cancer<br>•Endometrial Cancer	•Drug: CYH33
2	Combination ATR and PARP Inhibitor (CAPRI) Trial With AZD6738 and Olaparib in Recurrent Ovarian Cancer	Recruiting	No Results Available	•High Grade Serous Carcinoma	•Drug: Olaparib Pill<br>•Drug: AZD6738
3	PARP Inhibitor With 177Lu-DOTA-Octreotate PRRT in Patients With Neuroendocrine Tumours	Recruiting	No Results Available	•Neuroendocrine Tumors	•Drug: Talazoparib
4	Study of RP-3500 With Niraparib or Olaparib in Advanced Solid Tumors	Recruiting	No Results Available	•Advanced Solid Tumor, Adult	•Drug: RP-3500
5	Study to Assess the Safety and Tolerability of a PARP Inhibitor in Combination With Carboplatin and/or Paclitaxel	Active, not recruiting	No Results Available	•Triple Negative Metastatic Breast Cancer<br>•Advanced Ovarian Cancer<br>•Carboplatin<br>•Paclitaxel	•Drug: KU-0059436 (AZD2281) (PARP inhibitor)<br>•Drug: Carboplatin<br>•Drug: Paclitaxel<br>•Drug: Paclitaxel + Carboplatin
6	EP0057 in Combination With Olaparib in Advanced Ovarian Cancer	Recruiting	No Results Available	•Ovarian Cancer	•Drug: EP0057<br>•Drug: Olaparib tablets
7	A Study of AK112 Combined With PARP Inhibitor in the Treatment of Recurrent Ovarian Cancer	Recruiting	No Results Available	•Ovarian Neoplasms<br>•Recurrent Ovarian Carcinoma<br>•Relapsed Ovarian Cancer<br>•Ovarian Cancer	•Drug: AK112 low dose<br>•Drug: AK112 medium dose<br>•Drug: AK112 high dose
8	Study to Evaluate IMP9064 as a Monotherapy or in Combination in Patients With Advanced Solid Tumors	Recruiting	No Results Available	•Solid Tumor<br>•Advanced Solid Tumor	•Drug: IMP9064

	Title	Status	Study Results	Conditions	Interventions
9	A Phase II Clinical Trial Evaluating the Combination of Olaparib and Temozolomide for the Treatment of Advanced Uterine Leiomyosarcoma	Active, not recruiting	No Results Available	• Stage III Uterine Corpus Leiomyosarcoma AJCC v8<br>• Stage IIIA Uterine Corpus Leiomyosarcoma AJCC v8<br>• Stage IIIB Uterine Corpus Leiomyosarcoma AJCC v8<br>• Stage IIIC Uterine Corpus Leiomyosarcoma AJCC v8<br>• Stage IV Uterine Corpus Leiomyosarcoma AJCC v8<br>• Stage IVA Uterine Corpus Leiomyosarcoma AJCC v8<br>• Stage IVB Uterine Corpus Leiomyosarcoma AJCC v8	• Drug: Olaparib<br>• Drug: Temozolomide
10	Efficacy and Safety of the Combination of Rucaparib (PARP Inhibitor) and Atezolizumab (Anti-PD-L1 Antibody) in Patients With DNA Repair-deficient or Platinum-sensitive Solid Tumors	Recruiting	No Results Available	• Solid Tumor	• Drug: Atezolizumab<br>• Drug: Rucaparib
11	Study to Evaluate Sacituzumab Govitecan in Combination With Talazoparib in Patients With Metastatic Breast Cancer.	Recruiting	No Results Available	• Breast Cancer	• Drug: Talazoparib<br>• Drug: Sacituzumab Govitecan
12	Talazoparib in Combination With Belinostat for Metastatic Breast Cancer, Metastatic Castration Resistant Prostate Cancer, and Metastatic Ovarian Cancer	Recruiting	No Results Available	• Metastatic Breast Cancer<br>• Metastatic Castration-resistant Prostate Cancer<br>• Metastatic Ovarian Carcinoma	• Drug: Talazoparib<br>• Drug: Belinostat
13	Niraparib in Combination With Trastuzumab in Metastatic HER2+ Breast Cancer	Recruiting	No Results Available	• Metastatic Breast Cancer<br>• HER2 Positive Breast Carcinoma	• Drug: Niraparib<br>• Drug: Trastuzumab
14	Rucaparib in Combination With Nivolumab in Patients With Advanced or Metastatic Biliary Tract Cancer Following Platinum Therapy	Recruiting	No Results Available	• Biliary Tract Cancer	• Drug: Rucaparib<br>• Drug: Nivolumab
15	Study in Patients With SCLC of Veliparib in Combination With Topotecan	Recruiting	No Results Available	• Small Cell Lung Cancer SCLC	• Drug: veliparib<br>• Drug: Topotecan

	Title	Status	Study Results	Conditions	Interventions
16	Study of AZD5305 as Monotherapy and in Combination With Anti-cancer Agents in Patients With Advanced Solid Malignancies	Recruiting	No Results Available	•Ovarian Cancer<br>•Breast Cancer<br>•Pancreatic Cancer<br>•Prostate Cancer<br>•Additional Indications Below for Module 4 and 5<br>•Non-small Cell Lung Cancer<br>•Small Cell Lung Cancer<br>•Colorectal Cancer<br>•Bladder Cancer<br>•Gastric Cancer<br>•and 3 more	•Drug: AZD5305<br>•Drug: Paclitaxel<br>•Drug: Carboplatin<br>•Drug: T- Dxd<br>•Drug: Dato-DXd
17	ATr Inhibitor in Combination With Olaparib in Gynaecological Cancers With ARId1A Loss or no Loss	Recruiting	No Results Available	•Gynaecological Cancers	•Drug: AZD6738<br>•Drug: Olaparib
18	OLAParib COmbinations	Active, not recruiting	No Results Available	•Cancer	•Drug: AZD2281<br>•Drug: AZD5363<br>•Drug: AZD1775<br>•Drug: AZD6738
19	Anlotinib Combined With Dose-reduced Olaparib in Patients With Platinum-Sensitive Recurrent Ovarian Cancer	Recruiting	No Results Available	•Ovarian Cancer<br>•Ovarian and Fallopian Tube Cysts and Neoplasms<br>•Neoplasms by Site<br>•Neoplasms<br>•Genital Neoplasms, Female<br>•Urogenital Neoplasms<br>•Neoplasms, Glandular and Epithelial<br>•Neoplasms by Histologic Type<br>•Carcinoma, Ovarian Epithelial<br>•Ovarian Diseases<br>•and 9 more	•Drug: Anlotinib<br>•Drug: Olaparib

	Title	Status	Study Results	Conditions	Interventions
20	ZEN003694 Combined With Talazoparib in Patients With Recurrent Ovarian Cancer	Recruiting	No Results Available	• Ovarian Cancer<br>• Peritoneal Cancer<br>• Fallopian Tube Cancer	• Drug: ZEN003694<br>• Drug: Talazoparib
21	Pembrolizumab in Combination With Olaparib in Advanced BRCA-mutated or HDR-defect Breast Cancer	Recruiting	No Results Available	• Breast Cancer	• Drug: Pembrolizumab<br>• Drug: Olaparib
22	Pembrolizumab and Olaparib in Cervical Cancer Patients	Recruiting	No Results Available	• Cervical Cancer<br>• Cervical Carcinoma	• Drug: pembrolizumab<br>• Drug: olaparib
23	Study of RP-3500 in Advanced Solid Tumors	Recruiting	No Results Available	• Advanced Solid Tumor	• Drug: RP-3500<br>• Drug: Talazoparib<br>• Drug: Gemcitabine Injection [Gemzar]
24	IMP4297 in Combination With Temozolomide in Patients With Advanced Solid Tumors and Small Cell Lung Cancer	Recruiting	No Results Available	• Advanced Solid Tumours<br>• Small Cell Lung Cancer	• Drug: IMP4297
25	Testing the Combination of the Anti-cancer Drugs Copanlisib, Olaparib, and MEDI4736 (Durvalumab) in Patients With Advanced Solid Tumors With Selected Mutations	Recruiting	No Results Available	• Advanced Malignant Solid Neoplasm<br>• Metastatic Malignant Solid Neoplasm<br>• Unresectable Malignant Solid Neoplasm	• Drug: Copanlisib Hydrochloride<br>• Biological: Durvalumab<br>• Drug: Olaparib
26	ATR Inhibitor BAY 1895344 Plus Niraparib Phase 1b Study in Advanced Solid Tumors and Ovarian Cancer	Recruiting	No Results Available	• Advanced Solid Tumors (Excluding Prostate Cancer)<br>• Ovarian Cancer	• Drug: BAY1895344<br>• Drug: Niraparib

	Title	Status	Study Results	Conditions	Interventions
27	Comparison of Standard of Care Treatment With a Triplet Combination of Targeted Immunotherapeutic Agents	Recruiting	No Results Available	• Fallopian Tube Mucinous Adenocarcinoma<br>• Ovarian Seromucinous Carcinoma<br>• Platinum-Refractory Fallopian Tube Carcinoma<br>• Platinum-Refractory Ovarian Carcinoma<br>• Platinum-Refractory Primary Peritoneal Carcinoma<br>• Recurrent Fallopian Tube Clear Cell Adenocarcinoma<br>• Recurrent Fallopian Tube Endometrioid Adenocarcinoma<br>• Recurrent Fallopian Tube High Grade Serous Adenocarcinoma<br>• Recurrent Fallopian Tube Mucinous Adenocarcinoma<br>• Recurrent Fallopian Tube Transitional Cell Carcinoma<br>• and 40 more	• Drug: Cediranib Maleate<br>• Biological: Durvalumab<br>• Drug: Olaparib<br>• Drug: Paclitaxel<br>• Drug: Pegylated Liposomal Doxorubicin Hydrochloride<br>• Drug: Topotecan Hydrochloride
28	Testing Two Oral Drugs Combination (Cediranib and Olaparib) Compared to a Single Drug (Olaparib) for Men With Advanced Prostate Cancer	Active, not recruiting	No Results Available	• Advanced Prostate Adenocarcinoma With Neuroendocrine Differentiation<br>• Castration-Resistant Prostate Carcinoma<br>• Metastatic Prostate Adenocarcinoma With Neuroendocrine Differentiation<br>• Metastatic Prostate Carcinoma<br>• Prostate Adenocarcinoma With Neuroendocrine Differentiation<br>• Stage IV Prostate Adenocarcinoma AJCC v7	• Drug: Cediranib<br>• Drug: Olaparib

	Title	Status	Study Results	Conditions	Interventions
29	Testing the Combination of Anti-Cancer Drugs Talazoparib and Temozolomide in Patients >= 18 Years Old With Advanced Stage Rare Cancers, RARE 2 Study	Recruiting	No Results Available	• Adrenal Gland Pheochromocytoma<br>• Hematopoietic and Lymphoid Cell Neoplasm<br>• Malignant Solid Neoplasm<br>• Paraganglioma	• Drug: Talazoparib<br>• Drug: Temozolomide
30	Olaparib in Combination With Vorinostat in Patients With Relapsed/Refractory and/or Metastatic Breast Cancer	Recruiting	No Results Available	• Breast Cancer Metastatic<br>• Breast Cancer	• Drug: Olaparib<br>• Drug: Vorinostat
31	Testing the Addition of the Anti-Cancer Drug Talazoparib to the Combination of Carboplatin and Paclitaxel for the Treatment of Advanced Cancer	Active, not recruiting	No Results Available	• Advanced Malignant Solid Neoplasm<br>• Metastatic Malignant Solid Neoplasm<br>• Unresectable Malignant Solid Neoplasm	• Drug: Carboplatin<br>• Drug: Paclitaxel<br>• Drug: Talazoparib
32	BGB-290 and Temozolomide in Treating Isocitrate Dehydrogenase (IDH)1/2-Mutant Grade I-IV Gliomas	Recruiting	No Results Available	• Glioblastoma<br>• IDH1 Gene Mutation<br>• IDH2 Gene Mutation<br>• Low Grade Glioma<br>• Malignant Glioma<br>• Recurrent Glioblastoma<br>• Recurrent WHO Grade II Glioma<br>• Recurrent WHO Grade III Glioma<br>• WHO Grade II Glioma<br>• WHO Grade III Glioma	• Drug: PARP Inhibitor BGB-290<br>• Drug: Temozolomide
33	Pulmonary Toxicities Related to PARP Inhibitors (PulmonaRIB)	Recruiting	No Results Available	• Cancer	• Drug: olaparib, rucaparib, niraparib, talazoparib, veliparib, pamiparib
34	A Phase I/II Study of Sacituzumab Govitecan Plus Berzosertib in Small Cell Lung Cancer and Homologous Recombination-Deficient Cancers Resistant to PARP Inhibitors	Recruiting	No Results Available	• HRD Cancer<br>• SCLC<br>• Advanced Solid Tumors	• Drug: Berzosertib<br>• Drug: Sacituzumab Govitecan
35	A Study of ZEN003694 and Talazoparib in Patients With Triple Negative Breast Cancer	Active, not recruiting	No Results Available	• Triple Negative Breast Cancer	• Drug: ZEN003694<br>• Drug: Talazoparib

	Title	Status	Study Results	Conditions	Interventions
36	177Lu-DOTA-TATE and Olaparib in Somatostatin Receptor Positive Tumours	Recruiting	No Results Available	• Clinical Trial, Phase I<br>• Neuroendocrine Tumors<br>• Thymoma<br>• Mesothelioma	• Drug: 177Lu-DOTA-TATE + olaparib
37	Phase I/II Study of the Anti-Programmed Death Ligand-1 Durvalumab Antibody (MEDI4736) in Combination With Olaparib and/or Cediranib for Advanced Solid Tumors and Advanced or Recurrent Ovarian, Triple Negative Breast, Lung, Prostate and Colorectal Can...	Recruiting	No Results Available	• Colorectal Neoplasms<br>• Breast Neoplasms	• Drug: Olaparib<br>• Drug: Cediranib<br>• Drug: Durvalumab
38	A Trial of Pamiparib With Tislelizumab in Patients With Advanced Tumours With Homologous Recombination Repair Defects	Recruiting	No Results Available	• Cancer	• Drug: Pamiparib<br>• Drug: Tislelizumab
39	PLX038 (PEGylated SN38) and Rucaparib in Solid Tumors and Small Cell Cancers	Recruiting	No Results Available	• Small Cell Lung Cancer<br>• Extra-Pulmonary Small Cell Carcinomas	• Drug: PLX038<br>• Drug: Rucaparib
40	PARP-inhibition and CTLA-4 Blockade in BRCA-deficient Ovarian Cancer	Active, not recruiting	No Results Available	• Ovarian Cancer<br>• Fallopian Tube Cancer<br>• Peritoneal Neoplasms	• Drug: Olaparib<br>• Drug: Tremelimumab
41	Talazoparib and Palbociclib, Axitinib, or Crizotinib for the Treatment of Advanced or Metastatic Solid Tumors, TalaCom Trial	Recruiting	No Results Available	• Advanced Malignant Solid Neoplasm<br>• Metastatic Malignant Solid Neoplasm<br>• Recurrent Malignant Solid Neoplasm	• Drug: Axitinib<br>• Drug: Crizotinib<br>• Drug: Palbociclib Isethionate<br>• Drug: Talazoparib Tosylate

	Title	Status	Study Results	Conditions	Interventions
42	Veliparib and Temozolomide in Treating Patients With Acute Leukemia	Active, not recruiting	No Results Available	• Acute Lymphoblastic Leukemia<br>• Acute Myeloid Leukemia<br>• Acute Myeloid Leukemia Arising From Previous Myelodysplastic Syndrome<br>• Adult Acute Myeloid Leukemia With Inv(16)(p13.1q22); CBFB-MYH11<br>• Adult Acute Myeloid Leukemia With t(16;16)(p13.1;q22); CBFB-MYH11<br>• Adult Acute Myeloid Leukemia With t(8;21); (q22; q22.1); RUNX1-RUNX1T1<br>• Adult Acute Myeloid Leukemia With t(9;11)(p21.3;q23.3); MLLT3-MLL<br>• Adult Acute Promyelocytic Leukemia With PML-RARA<br>• Adult B Acute Lymphoblastic Leukemia<br>• Adult B Acute Lymphoblastic Leukemia With t(9;22)(q34.1;q11.2); BCR-ABL1<br>• and 6 more	• Other: Laboratory Biomarker Analysis<br>• Other: Pharmacological Study<br>• Drug: Temozolomide<br>• Drug: Veliparib
43	Ph I/II Study of NMS-03305293+TMZ in Adult Patients With Recurrent Glioblastoma	Recruiting	No Results Available	• Glioblastoma<br>• Diffuse Glioma	• Drug: NMS-03305293 + TMZ<br>• Drug: Lomustine
44	Niraparib in Combination With Osimertinib in EGFR-Mutated Advanced Lung Cancer	Recruiting	No Results Available	• Lung Cancer	• Drug: Niraparib<br>• Drug: Osimertinib
45	Induction and Maintenance Treatment With PARP Inhibitor and Immunotherapy in HPV-negative HNSCC	Recruiting	No Results Available	• Head and Neck Squamous Cell Carcinoma	• Drug: Niraparib
46	Durvalumab and Olaparib in Metastatic or Recurrent Endometrial Cancer	Active, not recruiting	No Results Available	• Endometrial Neoplasms<br>• Uterine Neoplasms<br>• Endometrium Cancer	• Drug: PARP inhibitor and Anti-PD-L1

	Title	Status	Study Results	Conditions	Interventions
47	ABT-888 and Temozolomide for Metastatic Breast Cancer and BRCA1/2 Breast Cancer	Active, not recruiting	No Results Available	•Breast Cancer<br>•Metastatic Breast Cancer<br>•BRCA1 Gene Mutation<br>•brca2 Gene Mutation	•Drug: ABT-888<br>•Drug: temozolomide
48	Testing Maintenance Therapy for Small Cell Lung Cancer in Patients With SLFN11 Positive Biomarker	Recruiting	No Results Available	•Extensive Stage Lung Small Cell Carcinoma	•Drug: Atezolizumab<br>•Drug: Talazoparib
49	Phase II Multicenter Study of Durvalumab and Olaparib in Platinum tReated Advanced Triple Negative Breast Cancer (DORA)	Active, not recruiting	No Results Available	•Triple Negative Breast Cancer	•Drug: Olaparib Oral Product<br>•Drug: Olaparib Oral Product in combination with Durvalumab
50	Recurrent Ovarian CarcinoSarcoma Anti-pd-1 Niraparib	Recruiting	No Results Available	•Ovarian Carcinosarcoma<br>•Endometrial Carcinosarcoma	•Drug: Niraparib<br>•Combination Product: Niraparib + TSR-042 (Dostarlimab)<br>•Drug: Chemotherapy Drugs
51	Carboplatin-Paclitaxel-Bevacizumab vs Carbo-Pacli-Beva-Rucaparib vs Carbo-Pacli-Ruca, Selected According to HRD Status, in Patients With Advanced Ovarian, Primary Peritoneal and Fallopian Tube Cancer, Preceded by a Phase I Dose Escalation Study on Ruca-Beva Combination	Recruiting	No Results Available	•Advanced (Stage IIIB-C-IV) Ovarian, Primary Peritoneal and Fallopian Tube Cancer	•Drug: Carboplatin<br>•Drug: Paclitaxel<br>•Drug: Bevacizumab<br>•Drug: Rucaparib
52	OSE2101 Alone or in Combination With Pembrolizumab vs BSC in Patient With Platinum-sensitive Recurrent OC	Recruiting	No Results Available	•Platinum-sensitive Ovarian Cancer<br>•Relapsed Ovarian Cancer	•Drug: OSE2101<br>•Drug: Pembrolizumab 25 MG/ML [Keytruda]
53	Surgery and Niraparib in Secondary Recurrent Ovarian Cancer (SOC-3 Trial)	Recruiting	No Results Available	•Ovarian Cancer Recurrent<br>•Fallopian Tube Cancer<br>•Primary Peritoneal Carcinoma	•Procedure: Surgery<br>•Drug: carboplatin/taxane, carboplatin/gemcitabine, cisplatin/ gemcitabine, liposome doxorubicin/ carboplatin...<br>•Drug: Niraparib
54	Testing the Safety of Different Doses of Olaparib Given Radium-223 for Men With Advanced Prostate Cancer With Bone Metastasis	Recruiting	No Results Available	•Castration-Resistant Prostate Carcinoma<br>•Metastatic Prostate Adenocarcinoma<br>•Stage IVB Prostate Cancer AJCC v8	•Other: Laboratory Biomarker Analysis<br>•Drug: Olaparib<br>•Other: Quality-of-Life Assessment<br>•Radiation: Radium Ra 223 Dichloride

	Title	Status	Study Results	Conditions	Interventions
55	A Phase 3 Randomized, Placebo-controlled Trial of Carboplatin and Paclitaxel With or Without Veliparib (ABT-888) in HER2-negative Metastatic or Locally Advanced Unresectable BRCA-associated Breast Cancer	Active, not recruiting	Has Results	•Metastatic Breast Cancer	•Drug: Veliparib Placebo<br>•Drug: Veliparib<br>•Drug: Carboplatin<br>•Drug: Paclitaxel
56	Decitabine and Talazoparib in Untreated AML and R/R AML	Active, not recruiting	No Results Available	•Acute Myeloid Leukemia	•Drug: Decitabine<br>•Drug: talazoparib
57	A Phase 2 Trial of Fluzoparib Combined With Apatinib Versus Fluzoparib Monotherapy in Treatment With Relapsed Ovarian Cancer Patients	Recruiting	No Results Available	•Relapsed Ovarian Cancer	•Drug: Fluzoparib+Apatinib<br>•Drug: Fluzoparib
58	A Study to Evaluate the Efficacy and Safety of Novel Treatment Combinations in Participants With Ovarian Cancer	Recruiting	No Results Available	•Ovarian Neoplasms	•Drug: Niraparib<br>•Biological: TSR-042<br>•Biological: Bevacizumab<br>•Drug: Carboplatin<br>•Drug: Paclitaxel
59	Niraparib, Temozolomide and Atezolizumab in Treating Patients With Advanced Solid Tumors and Extensive-Stage Small Cell Lung Cancer With a Complete or Partial Response to Platinum-Based First-Line Chemotherapy	Recruiting	No Results Available	•Advanced Malignant Solid Neoplasm<br>•Extensive Stage Lung Small Cell Carcinoma<br>•Stage III Lung Cancer AJCC v8<br>•Stage IIIA Lung Cancer AJCC v8<br>•Stage IIIB Lung Cancer AJCC v8<br>•Stage IIIC Lung Cancer AJCC v8<br>•Stage IV Lung Cancer AJCC v8<br>•Stage IVA Lung Cancer AJCC v8<br>•Stage IVB Lung Cancer AJCC v8	•Biological: Atezolizumab<br>•Drug: Niraparib<br>•Other: Quality-of-Life Assessment<br>•Other: Questionnaire Administration<br>•Drug: Temozolomide
60	Radiation, Immunotherapy and PARP Inhibitor in Triple Negative Breast Cancer	Recruiting	No Results Available	•Breast Cancer<br>•Triple Negative Breast Cancer	•Drug: Niraparib<br>•Drug: Dostarlimab<br>•Radiation: Radiation therapy
61	Phase I/IIa Study of Concomitant Radiotherapy With Olaparib and Temozolomide in Unresectable High Grade Gliomas Patients	Recruiting	No Results Available	•Malignant Gliomas<br>•Radiotherapy<br>•PARP Inhibitor	•Drug: Olaparib<br>•Drug: Temozolomide (TMZ)<br>•Radiation: IMRT (Intensity Modulated Radiation Therapy)

	Title	Status	Study Results	Conditions	Interventions
62	Abemaciclib and Niraparib Before Surgery for the Treatment of Hormone Receptor Positive HER2 Negative Breast Cancer	Recruiting	No Results Available	•Anatomic Stage I Breast Cancer AJCC v8<br>•Anatomic Stage IA Breast Cancer AJCC v8<br>•Anatomic Stage IB Breast Cancer AJCC v8<br>•Anatomic Stage II Breast Cancer AJCC v8<br>•Anatomic Stage IIA Breast Cancer AJCC v8<br>•Anatomic Stage IIB Breast Cancer AJCC v8<br>•Anatomic Stage IIIA Breast Cancer AJCC v8<br>•HER2 Negative Breast Adenocarcinoma<br>•Hormone Receptor Positive Breast Adenocarcinoma<br>•Invasive Breast Carcinoma<br>•and 10 more	•Drug: Abemaciclib<br>•Drug: Niraparib Tosylate Monohydrate
63	Veliparib With Carboplatin and Paclitaxel and as Continuation Maintenance Therapy in Adults With Newly Diagnosed Stage III or IV, High-grade Serous, Epithelial Ovarian, Fallopian Tube, or Primary Peritoneal Cancer	Active, not recruiting	Has Results	•Ovarian Cancer<br>•Ovarian Neoplasm	•Drug: Veliparib<br>•Drug: Paclitaxel<br>•Drug: Carboplatin<br>•Other: Placebo to Veliparib

	Title	Status	Study Results	Conditions	Interventions
64	A Randomized Study of Olaparib or Placebo in Patients With Surgically Removed Pancreatic Cancer Who Have a BRCA1, BRCA2 or PALB2 Mutation, The APOLLO Trial	Recruiting	No Results Available	•Pancreatic Acinar Cell Carcinoma<br>•Pancreatic Adenosquamous Carcinoma<br>•Pancreatic Carcinoma<br>•Pancreatic Squamous Cell Carcinoma<br>•Resectable Pancreatic Acinar Cell Carcinoma<br>•Resectable Pancreatic Adenocarcinoma<br>•Resectable Pancreatic Adenosquamous Carcinoma<br>•Resectable Pancreatic Carcinoma<br>•Stage 0 Pancreatic Cancer AJCC v8<br>•Stage I Pancreatic Cancer AJCC v8<br>•and 5 more	•Drug: Olaparib<br>•Drug: Placebo Administration
65	NUV-868 as Monotherapy and in Combination With Olaparib or Enzalutamide in Adult Patients With Advanced Solid Tumors	Recruiting	No Results Available	•Advanced Solid Tumor<br>•Ovarian Cancer<br>•Ovary Cancer<br>•Cancer of Ovary<br>•Cancer of the Ovary<br>•Ovary Neoplasm<br>•Pancreatic Cancer<br>•Pancreas Cancer<br>•Cancer of Pancreas<br>•Cancer of the Pancreas<br>•and 17 more	•Drug: NUV-868<br>•Drug: Olaparib<br>•Drug: Enzalutamide
66	Study of Oral Rucaparib With Other Anticancer Agents in Metastatic Castration Resistant Prostate Cancer Patients (RAMP)	Active, not recruiting	No Results Available	•Metastatic Castration Resistant Prostate Cancer	•Drug: Rucaparib<br>•Drug: Enzalutamide<br>•Drug: Abiraterone

	Title	Status	Study Results	Conditions	Interventions
67	Phase II Study of Olaparib and Pembrolizumab in Advanced Melanoma With Homologous Recombination (HR) Mutation	Recruiting	No Results Available	•Metastatic Melanoma	•Drug: Olaparib<br>•Drug: Pembrolizumab
68	Targeted Agent Evaluation in Digestive Cancers in China Based on Molecular Characteristics	Recruiting	No Results Available	•Biliary Tract Neoplasms<br>•Gastric Cancer<br>•Esophageal Squamous Cell Carcinoma<br>•Colorectal Cancer<br>•Gastrointestinal Stromal Tumors<br>•Pancreatic Cancer<br>•Neuroendocrine Tumors<br>•Unknown Primary Cancer<br>•Digestive Cancer	•Drug: FGFR Inhibitor, IDH1 Inhibitor, HER2 Inhibitor, PARP Inhibitor, BRAF Inhibitor, MEK Inhibitor, ICIs, EGFR-TKIs, NTRK-TKI, and et. al.<br>•Drug: Other Therapy
69	BGB-290 and Temozolomide in Treating Patients With Recurrent Gliomas With IDH1/2 Mutations	Active, not recruiting	No Results Available	•IDH1 Mutation<br>•IDH2 Mutation<br>•Recurrent Glioblastoma<br>•Recurrent WHO Grade II Glioma<br>•Recurrent WHO Grade III Glioma	•Drug: PARP Inhibitor BGB-290<br>•Drug: Temozolomide<br>•Procedure: Therapeutic Conventional Surgery
70	NUVOLA TRIAL Open-label Multicentre Study	Recruiting	No Results Available	•High Grade Serous Ovarian Cancer	•Drug: Olaparib<br>•Drug: Paclitaxel<br>•Drug: Carboplatin

	Title	Status	Study Results	Conditions	Interventions
71	Cediranib Maleate and Olaparib in Treating Patients With Recurrent Ovarian, Fallopian Tube, or Peritoneal Cancer or Recurrent Triple-Negative Breast Cancer	Active, not recruiting	Has Results	•Ovarian Endometrioid Adenocarcinoma<br>•Ovarian High Grade Serous Adenocarcinoma<br>•Ovarian Serous Adenocarcinoma<br>•Ovarian Serous Surface Papillary Adenocarcinoma<br>•Primary Peritoneal Serous Adenocarcinoma<br>•Recurrent Breast Carcinoma<br>•Recurrent Fallopian Tube Carcinoma<br>•Recurrent Ovarian Carcinoma<br>•Recurrent Primary Peritoneal Carcinoma<br>•Triple-Negative Breast Carcinoma	•Drug: Cediranib Maleate<br>•Drug: Olaparib
72	Testing the Combination of DS-8201a and Olaparib in HER2-Expressing Cancers With Expansion in Patients With Endometrial Cancer	Recruiting	No Results Available	•Endometrial Serous Adenocarcinoma<br>•Metastatic Malignant Solid Neoplasm<br>•Unresectable Malignant Solid Neoplasm	•Drug: Olaparib<br>•Biological: Trastuzumab Deruxtecan
73	Talazoparib - Carboplatin for Recurrent High-grade Glioma With DDRd	Recruiting	No Results Available	•Recurrent Glioma<br>•Recurrent Glioblastoma<br>•Poly ADP Ribose Polymerase (PARP) Inhibitor<br>•PTEN Gene Inactivation<br>•IDH Mutation	•Drug: Talazoparib
74	Study to Compare the Efficacy and Safety of Olaparib When Given in Combination With Carboplatin and Paclitaxel, Compared With Carboplatin and Paclitaxel in Patients With Advanced Ovarian Cancer	Active, not recruiting	Has Results	•Ovarian Cancer	•Drug: olaparib<br>•Drug: paclitaxel<br>•Drug: carboplatin<br>•Drug: Drug: carboplatin

	Title	Status	Study Results	Conditions	Interventions
75	Olaparib, Durvalumab, and Tremelimumab in Treating Patients With Recurrent or Refractory Ovarian, Fallopian Tube or Primary Peritoneal Cancer With BRCA1 or BRCA2 Mutation	Active, not recruiting	No Results Available	•BRCA1 Gene Mutation<br>•BRCA2 Gene Mutation<br>•Ovarian Serous Adenocarcinoma<br>•Recurrent Fallopian Tube Carcinoma<br>•Recurrent Ovarian Carcinoma<br>•Recurrent Primary Peritoneal Carcinoma	•Biological: Durvalumab<br>•Other: Laboratory Biomarker Analysis<br>•Drug: Olaparib<br>•Biological: Tremelimumab
76	M1774 in Participants With Metastatic or Locally Advanced Unresectable Solid Tumors (DDRiver Solid Tumors 301)	Recruiting	No Results Available	•Metastatic or Locally Advanced Unresectable Solid Tumors	•Drug: M1774<br>•Drug: Niraparib
77	Olaparib and Durvalumab With Carboplatin, Etoposide, and/or Radiation Therapy for the Treatment of Extensive-Stage Small Cell Lung Cancer, PRIO Trial	Recruiting	No Results Available	•Extensive Stage Lung Small Cell Carcinoma<br>•Stage IV Lung Cancer AJCC v8<br>•Stage IVA Lung Cancer AJCC v8<br>•Stage IVB Lung Cancer AJCC v8	•Drug: Carboplatin<br>•Biological: Durvalumab<br>•Drug: Etoposide<br>•Drug: Olaparib<br>•Radiation: Radiation Therapy
78	Olaparib and Radiotherapy in Head and Neck Cancer	Active, not recruiting	No Results Available	•Laryngeal Cancer Stage II<br>•Laryngeal Cancer Stage III<br>•Carcinoma, Squamous Cell<br>•Head and Neck Neoplasms	•Radiation: radiotherapy<br>•Drug: Olaparib
79	A Study of ART4215 for the Treatment of Advanced or Metastatic Solid Tumors	Recruiting	No Results Available	•Advanced Cancer<br>•Metastatic Cancer<br>•Breast Cancer	•Drug: ART4215<br>•Drug: Talazoparib<br>•Drug: Niraparib
80	Trial of EP0057, a Nanoparticle Camptothecin With Olaparib in People With Relapsed/Refractory Small Cell Lung Cancer	Recruiting	No Results Available	•Urothelial Carcinoma<br>•Urothelial Cancer<br>•Lung Neoplasms<br>•Small Cell Lung Cancer<br>•Prostate Cancer	•Drug: EP0057<br>•Drug: olaparib

	Title	Status	Study Results	Conditions	Interventions
81	Study of Onivyde With Talazoparib or Temozolomide in Children With Recurrent Solid Tumors and Ewing Sarcoma	Active, not recruiting	No Results Available	• Recurrent Solid Tumor<br>• Recurrent Ewing Sarcoma<br>• Recurrent Hepatoblastoma<br>• Recurrent Malignant Germ Cell Tumor<br>• Recurrent Malignant Solid Neoplasm<br>• Recurrent Neuroblastoma<br>• Recurrent Osteosarcoma<br>• Recurrent Peripheral Primitive Neuroectodermal Tumor<br>• Recurrent Rhabdoid Tumor<br>• Recurrent Rhabdomyosarcoma<br>• and 12 more	• Drug: Onivyde<br>• Drug: Talazoparib<br>• Drug: Temozolomide
82	Study Evaluating the Efficacy of Maintenance Olaparib and Cediranib or Olaparib Alone in Ovarian Cancer Patients	Recruiting	No Results Available	• Ovarian Cancer	• Drug: Olaparib<br>• Drug: Cediranib
83	Study of Talazoparib With Enzalutamide in Men With DDR Gene Mutated mCSPC	Recruiting	No Results Available	• Prostate Cancer	• Drug: talazoparib plus enzalutamide<br>• Drug: Placebo plus enzalutamide
84	Efficacy Study of Olaparib With Paclitaxel Versus Paclitaxel in Gastric Cancer Patients	Active, not recruiting	Has Results	• Gastric Cancer	• Drug: olaparib<br>• Drug: paclitaxel<br>• Drug: Placebo
85	Alpelisib Plus Olaparib in Platinum-resistant/ Refractory, High-grade Serous Ovarian Cancer, With no Germline BRCA Mutation Detected	Recruiting	No Results Available	• Ovarian Cancer	• Drug: Alpelisib<br>• Drug: Olaparib<br>• Drug: Paclitaxel<br>• Drug: Pegylated liposomal doxorubicin (PLD)

	Title	Status	Study Results	Conditions	Interventions
86	A Phase 2 Study of Cediranib in Combination With Olaparib in Advanced Solid Tumors	Active, not recruiting	No Results Available	•Metastatic Lung Non-Small Cell Carcinoma<br>•Metastatic Lung Small Cell Carcinoma<br>•Metastatic Pancreatic Adenocarcinoma<br>•Metastatic Triple-Negative Breast Carcinoma<br>•Pancreatic Ductal Adenocarcinoma<br>•Stage III Breast Cancer AJCC v7<br>•Stage III Lung Non-Small Cell Cancer AJCC v7<br>•Stage III Lung Small Cell Carcinoma AJCC v7<br>•Stage III Pancreatic Cancer AJCC v6 and v7<br>•Stage IIIA Breast Cancer AJCC v7<br>•and 15 more	•Other: 18F-Fluoromisonidazole<br>•Drug: Cediranib Maleate<br>•Other: Laboratory Biomarker Analysis<br>•Drug: Olaparib<br>•Procedure: Positron Emission Tomography
87	Niraparib + Ipilimumab or Nivolumab in Progression Free Pancreatic Adenocarcinoma After Platinum-Based Chemotherapy	Active, not recruiting	No Results Available	•Pancreatic Adenocarcinoma	•Drug: Niraparib + Nivolumab<br>•Drug: Niraparib + Ipilimumab
88	Hyperthermia and Olaparib in Treating Breast Cancer Patients With Chest Wall Recurrences	Active, not recruiting	No Results Available	•Metastatic Malignant Neoplasm in the Chest Wall<br>•Recurrent Breast Carcinoma	•Drug: Olaparib<br>•Procedure: Hyperthermia Treatment<br>•Other: Questionnaire Administration<br>•Other: Quality-of-Life Assessment

	Title	Status	Study Results	Conditions	Interventions
89	Olaparib and Ramucirumab in Treating Patients With Metastatic or Locally Recurrent Gastric or Gastroesophageal Junction Cancer That Cannot Be Removed by Surgery	Active, not recruiting	No Results Available	•Metastatic Esophageal Carcinoma<br>•Metastatic Gastric Carcinoma<br>•Metastatic Gastroesophageal Junction Adenocarcinoma<br>•Recurrent Esophageal Carcinoma<br>•Recurrent Gastric Carcinoma<br>•Recurrent Gastroesophageal Junction Adenocarcinoma<br>•Stage III Esophageal Cancer AJCC v7<br>•Stage III Gastric Cancer AJCC v7<br>•Stage IIIA Esophageal Cancer AJCC v7<br>•Stage IIIA Gastric Cancer AJCC v7<br>•and 9 more	•Drug: Olaparib<br>•Biological: Ramucirumab
90	Liposomal Irinotecan, Fluorouracil, Leucovorin Calcium, and Rucaparib in Treating Patients With Metastatic Pancreatic, Colorectal, Gastroesophageal, or Biliary Cancer	Active, not recruiting	No Results Available	•Metastatic Biliary Tract Carcinoma<br>•Metastatic Colorectal Carcinoma<br>•Metastatic Gastroesophageal Junction Adenocarcinoma<br>•Metastatic Malignant Digestive System Neoplasm<br>•Metastatic Pancreatic Adenocarcinoma<br>•Stage IV Colorectal Cancer AJCC v7<br>•Stage IV Pancreatic Cancer AJCC v6 and v7<br>•Stage IVA Colorectal Cancer AJCC v7<br>•Stage IVB Colorectal Cancer AJCC v7	•Drug: Fluorouracil<br>•Drug: Irinotecan Sucrosofate<br>•Other: Laboratory Biomarker Analysis<br>•Drug: Leucovorin Calcium<br>•Drug: Rucaparib

	Title	Status	Study Results	Conditions	Interventions
91	Durvalumab and Olaparib for the Treatment of Prostate Cancer in Men Predicted to Have a High Neoantigen Load	Recruiting	No Results Available	• Biochemically Recurrent Prostate Carcinoma<br>• Prostate Adenocarcinoma	• Biological: Durvalumab<br>• Drug: Olaparib<br>• Other: Quality-of-Life Assessment<br>• Other: Questionnaire Administration
92	Olaparib and Durvalumab in Treating Patients With Metastatic Triple Negative Breast Cancer	Recruiting	No Results Available	• Anatomic Stage IV Breast Cancer AJCC v8<br>• Metastatic Triple-Negative Breast Carcinoma<br>• Prognostic Stage IV Breast Cancer AJCC v8	• Biological: Durvalumab<br>• Drug: Olaparib
93	Testing the Combination of Olaparib and Durvalumab, Cediranib and Durvalumab, Olaparib and Capivasertib, and Cediranib Alone in Recurrent or Refractory Endometrial Cancer Following the Earlier Phase of the Study That Tested Olaparib and Cediranib in Comparison to Cediranib Alone, and Olaparib Alone	Recruiting	No Results Available	• Endometrial Undifferentiated Carcinoma<br>• Endometrioid Adenocarcinoma<br>• Recurrent Endometrial Serous Adenocarcinoma<br>• Recurrent Uterine Corpus Cancer<br>• Stage IV Uterine Corpus Cancer AJCC v7<br>• Stage IVA Uterine Corpus Cancer AJCC v7<br>• Stage IVB Uterine Corpus Cancer AJCC v7	• Drug: Capivasertib<br>• Drug: Cediranib<br>• Drug: Cediranib Maleate<br>• Biological: Durvalumab<br>• Drug: Olaparib
94	FUSCC Refractory TNBC Umbrella (FUTURE)	Recruiting	No Results Available	• Triple-negative Breast Cancer	• Drug: Pyrotinib with Capecitabine<br>• Drug: AR inhibitor combined with everolimus(B1) or CDK4/6 inhibitor(B2),or EZH2 inhibitor (B4)<br>• Drug: anti PD-1 with nab-paclitaxel<br>• Drug: PARP inhibitor included therapy<br>• Drug: BLIS with anti-VEGFR included therapy<br>• Drug: MES with anti-VEGFR included therapy<br>• Drug: mTOR inhibitor with nab-paclitaxel

	Title	Status	Study Results	Conditions	Interventions
95	Talazoparib and Low-Dose Temozolomide in Treating Participants With Relapsed or Refractory Extensive-Stage Small Cell Lung Cancer	Active, not recruiting	No Results Available	• Recurrent Extensive Stage Small Cell Lung Carcinoma<br>• Refractory Extensive Stage Small Cell Lung Carcinoma	• Drug: Talazoparib<br>• Drug: Temozolomide
96	Javelin Parp Medley: Avelumab Plus Talazoparib In Locally Advanced Or Metastatic Solid Tumors	Active, not recruiting	No Results Available	• Avelumab in Combination With Talazoparib Will be Investigated in Patients With Locally Advanced (Primary or Recurrent) or Metastatic Solid Tumors	• Drug: Avelumab Phase 1b<br>• Drug: Talazoparib Phase 1b<br>• Drug: Avelumab Phase 2<br>• Drug: Talazoparib Phase 2
97	Serial Measurements of Molecular and Architectural Responses to Therapy (SMMART) PRIME Trial	Recruiting	No Results Available	• Accelerated Phase Chronic Myelogenous Leukemia, BCR-ABL1 Positive<br>• Anatomic Stage IV Breast Cancer AJCC v8<br>• Anemia<br>• Ann Arbor Stage III Hodgkin Lymphoma<br>• Ann Arbor Stage III Non-Hodgkin Lymphoma<br>• Ann Arbor Stage IIIA Hodgkin Lymphoma<br>• Ann Arbor Stage IIIB Hodgkin Lymphoma<br>• Ann Arbor Stage IV Hodgkin Lymphoma<br>• Ann Arbor Stage IV Non-Hodgkin Lymphoma<br>• Ann Arbor Stage IVA Hodgkin Lymphoma<br>• and 49 more	• Drug: Abemaciclib<br>• Drug: Abiraterone<br>• Drug: Afatinib<br>• Biological: Bevacizumab<br>• Drug: Bicalutamide<br>• Procedure: Biospecimen Collection<br>• Drug: Bortezomib<br>• Drug: Cabazitaxel<br>• Drug: Cabozantinib<br>• Drug: Capecitabine<br>• and 46 more
98	A Study of Rucaparib Versus Physician's Choice of Therapy in Patients With Metastatic Castration-resistant Prostate Cancer and Homologous Recombination Gene Deficiency	Active, not recruiting	No Results Available	• Metastatic Castration Resistant Prostate Cancer	• Drug: Rucaparib<br>• Drug: Abiraterone acetate or Enzalutamide or Docetaxel

	Title	Status	Study Results	Conditions	Interventions
99	Testing the Combination of Cediranib and Olaparib in Comparison to Each Drug Alone or Other Chemotherapy in Recurrent Platinum-Resistant Ovarian Cancer	Active, not recruiting	No Results Available	• Fallopian Tube Clear Cell Adenocarcinoma<br>• Fallopian Tube Endometrioid Adenocarcinoma<br>• Fallopian Tube Serous Adenocarcinoma<br>• Fallopian Tube Transitional Cell Carcinoma<br>• Fallopian Tube Undifferentiated Carcinoma<br>• Ovarian Clear Cell Adenocarcinoma<br>• Ovarian Endometrioid Adenocarcinoma<br>• Ovarian Seromucinous Carcinoma<br>• Ovarian Serous Adenocarcinoma<br>• Ovarian Transitional Cell Carcinoma<br>• and 5 more	• Drug: Cediranib<br>• Drug: Cediranib Maleate<br>• Drug: Olaparib<br>• Drug: Paclitaxel<br>• Drug: Pegylated Liposomal Doxorubicin Hydrochloride<br>• Other: Questionnaire Administration<br>• Drug: Topotecan<br>• Drug: Topotecan Hydrochloride
100	Paclitaxel, Pembrolizumab and Olaparib in Previously Treated Advanced Gastric Adenocarcinoma	Recruiting	No Results Available	• Advanced Gastric Adenocarcinoma	• Drug: Paclitaxel<br>• Drug: Olaparib<br>• Drug: Pembrolizumab

11 additional studies not shown
